# Disease clearance in ulcerative colitis: Is the ultimate therapeutic target?

**DOI:** 10.1002/ueg2.12436

**Published:** 2023-07-03

**Authors:** Ferdinando D’Amico, Laurent Peyrin‐Biroulet, Silvio Danese

**Affiliations:** ^1^ Gastroenterology and Endoscopy IRCCS Ospedale San Raffaele and Vita‐Salute San Raffaele University Milan Italy; ^2^ Department of Biomedical Sciences Humanitas University Milan Italy; ^3^ Department of Gastroenterology Nancy University Hospital Vandœuvre‐lès‐Nancy France; ^4^ INSERM NGERE University of Lorraine Nancy France; ^5^ INFINY Institute Nancy University Hospital Vandœuvre‐lès‐Nancy France; ^6^ FHU‐CURE Nancy University Hospital Vandœuvre‐lès‐Nancy France; ^7^ Groupe Hospitalier Privé Ambroise Paré – Hartmann Paris IBD Center Neuilly‐sur‐Seine France; ^8^ Division of Gastroenterology and Hepatology McGill University Health Centre Montreal Quebec Canada

**Keywords:** gastroenterology, IBD, inflammation, inflammatory bowel disease, ulcerative colitis

Treatment targets for ulcerative colitis (UC) have evolved considerably in recent years from symptom control to endoscopic remission.^1^ However, patients with endoscopic remission but persistent histological disease activity have a worse prognosis, thus suggesting that histological activity should be considered as an additional target.^2^ From the attempt to achieve ever deeper remission, the concept of disease clearance has been hypothesized.^3^ It is defined as simultaneous clinical, endoscopic, and histologic remission. Wolfgang and colleagues conducted a post‐hoc analysis of 4 randomised phase 3 trials to investigate the rates of disease clearance in 860 patients with mild‐to‐moderate UC treated with different doses of mesalazine as induction therapy. Interestingly, 20% of patients experienced disease clearance after 8 weeks, supporting that this composite endpoint is achievable in daily clinical practice. Another multicenter retrospective study including nearly 500 UC patients confirmed that disease clearance was reported in approximately 20% of cases after any induction therapy.^4^ Interestingly, the achievement of disease clearance was associated with a significant reduction in the risk of hospitalization and surgery during a median follow‐up of 2 years. These data demonstrate how patients with disease clearance have a better prognosis. Moreover, reaching this composite endpoint could allow to reduce the UC‐related complications and the healthcare indirect costs. The term disease clearance has been coined in dermatology and is associated with a significant improvement in patients' quality of life. It is also used to directly compare drug efficacy.^5^ In psoriasis, it is defined as total disappearance of skin lesions and is measured using a validated score, the Psoriasis Area and Severity Index 100.^6^ Similarly, the Eczema Area Severity Index improvement = 100% (EASI‐100) defines disease clearance in patients with atopic dermatitis.^7^ This attempt to achieve a total and lasting remission is common to several immune‐mediated disorders. To date, disease clearance also plays a role in Crohn's disease with transmural remission recognized as the main driver.^8^ Disease clearance is increasingly investigated as an exploratory endpoint or assessed via post‐hoc analyses in phase 3‐4 trials (NCT03259334).^9^ However, there are some issues that limit its wide use. Firstly, a validated and commonly accepted definition in UC is missing. Of course, disease clearance requires strict criteria proving the absence of disease. The use of validated clinical, endoscopic, and histologic scores could certainly facilitate its use and approval. A definition approved by the International Organization for the Study of Inflammatory Bowel Diseases will soon be available allowing to standardize disease clearance evaluation. Secondly, although there is evidence that disease clearance is an achievable target, the proportion of patients experiencing this composite outcome is limited. Several new molecules have recently been approved for the management of moderate‐to‐severe UC, such as selective Janus kinases inhibitors (filgotinib and upadacitinib) and sphingosine‐1‐phosphate modulator (ozanimod).^10^ However, even with these new drugs, rates of remission greater than 50% are not obtained and a loss of response to the drug after an initial improvement is frequent. There are several strategies to overcome this so‐called “therapeutic ceiling” including an early diagnosis and consequently an early treatment and the use of dual therapy, defined as the combination of biological drugs and/or small molecules.^11^ There is evidence supporting the efficacy and safety of dual therapy in UC, but unfortunately, no data are available regarding the rate of disease clearance in this setting.^12^ Furthermore, data on disease clearance from randomized clinical trials are scarce. Recently, preliminary data from the VERDICT trial were published.^13^ VERDICT is the first trial specifically designed to determine whether a treatment target approach based on disease clearance is superior to symptom control alone or symptom control associated with endoscopic remission. It will provide important guidance for the management of patients with UC, assess the weight of each component of disease clearance, and represent the starting point for future disease‐modification trials. Of note, this tight monitoring could allow early identification of disease recurrences and timely escalation of medical therapy, ensuring the best patients' quality of life is maintained. Finally, it is necessary to underline how the concept of disease clearance could evolve in the near future on the basis of new evidence and a greater understanding of the pathophysiological mechanisms of UC. Indeed, there are transcriptional signatures implicated in the expression of inflammatory molecules, thus suggesting the presence of potential molecular targets.^14^ If these data are confirmed, it is legitimate to hypothesize a molecular remission as a therapeutic target to achieve a deeper remission close to being considered curative.

## AUTHOR CONTRIBUTIONS

Ferdinando D’Amico conceived the article. Laurent Peyrin‐Biroulet and Silvio Danese critically reviewed the content of the paper and supervised the project. The manuscript was approved by all authors.

## CONFLICT OF INTEREST STATEMENT

F. D’Amico has served as a speaker for Sandoz, Janssen, Galapagos, and Omega Pharma; he also served as an advisory board member for Galapagos, Abbvie and Nestlè. L. Peyrin‐Biroulet received fees from AbbVie, Adacyte, Alimentiv, Alma Bio Therapeutics, Amgen, Applied Molecular Transport, Arena, Biogen, BMS, Celltrion, CONNECT Biopharm, Cytoki Pharma, Enthera, Ferring, Fresenius Kabi, Galapagos, Genentech, Gilead, Gossamer Bio, GSK, HAC‐Pharma, IAG Image Analysis, Index Pharmaceuticals, Inotrem, Janssen, Lilly, Medac, Mopac, Morphic, MSD, Norgine, Nordic Pharma, Novartis, OM Pharma, ONO Pharma, OSE Immunotherapeutics, Pandion Therapeutics, Par’Immune, Pfizer, Prometheus, Protagonist, Roche, Sanofi, Sandoz, Takeda, Theravance, Thermo Fisher, Tigenix, Tillots, Viatris, Vifor, Ysopia, Abivax, Samsung, Ventyx, Roivant, Vectivbio. S. Danese has served as a speaker, consultant, and advisory board member for Schering‐Plough, AbbVie, Actelion, Alphawasserman, AstraZeneca, Cellerix, Cosmo Pharmaceuticals, Ferring, Genentech, Grunenthal, Johnson and Johnson, Millenium Takeda, MSD, Nikkiso Europe GmbH, Novo Nordisk, Nycomed, Pfizer, Pharmacosmos, UCB Pharma and Vifor.

## Data Availability

No new data were generated or analyzed in support of this research.

## References

[1] Peyrin‐Biroulet L , Sandborn W , Sands BE , Reinisch W , Bemelman W , Bryant RV , et al. Selecting Therapeutic Targets in Inflammatory Bowel Disease (STRIDE): determining therapeutic goals for treat‐to‐target. Am J Gastroenterol. 2015;110(9):1324–1338. 10.1038/ajg.2015.233 26303131

[2] Turner D , Ricciuto A , Lewis A , D’Amico F , Dhaliwal J , Griffiths AM , et al. STRIDE‐II: an update on the Selecting Therapeutic Targets in Inflammatory Bowel Disease (STRIDE) initiative of the International Organization for the Study of IBD (IOIBD): determining therapeutic goals for treat‐to‐target strategies in IBD. Gastroenterology. 2021;160(5):1570–1583. 10.1053/j.gastro.2020.12.031 33359090

[3] Danese S , Roda G , Peyrin‐Biroulet L . Evolving therapeutic goals in ulcerative colitis: towards disease clearance. Nat Rev Gastroenterol Hepatol. 2020;17:1–2. 10.1038/s41575-019-0211-1 31520081

[4] D’Amico F , Fiorino G , Solitano V , Massarini E , Guillo L , Allocca M , et al. Ulcerative colitis: impact of early disease clearance on long‐term outcomes – a multicenter cohort study. Uni. Eur Gastroenterol J. 2022;10(7):775–782. 10.1002/ueg2.12288 PMC9486490 36107109

[5] Reich K , Gooderham M , Thaçi D , Crowley JJ , Ryan C , Krueger JG , et al. Risankizumab compared with adalimumab in patients with moderate‐to‐severe plaque psoriasis (IMMvent): a randomised, double‐blind, active‐comparator‐controlled phase 3 trial. Lancet. 2019;394(10198):576–586. 10.1016/s0140-6736(19)30952-3 31280967

[6] Reich K , Warren RB , Lebwohl M , Gooderham M , Strober B , Langley RG , et al. Bimekizumab versus Secukinumab in plaque psoriasis. N Engl J Med. 2021;385(2):142–152. 10.1056/nejmoa2102383 33891380

[7] Blauvelt A , Silverberg JI , Calimlim BM , Liu Y , Platt A , Thyssen JP . 355 Efficacy of upadacitinib for moderate‐to‐severe atopic dermatitis: analysis of time spent in skin clearance response states from the Measure Up 1, Measure Up 2 and Heads Up studies. Br J Dermatol. 2023;188(Supplement_2):ljac140.047. 10.1093/bjd/ljac140.047

[8] Queiroz NSF , Cheifetz AS , Papamichael K . Going beyond a treat‐to‐target to a treat‐to‐clear and a treat‐to‐trough therapeutic Strategy in Patients with Inflammatory Bowel Disease? J Crohn’s Colitis. 2023;17(6):825–826. 10.1093/ecco-jcc/jjad014 36827471

[9] Danese S , Schreiber S , Loftus E, Jr , Colombel JF , Peyrin‐Biroulet L , Agboton C , et al. P271 evolving targets in ulcerative colitis: defining disease clearance in the VARSITY study. J Crohn’s Colitis. 2021;15(Supplement_1):S305. 10.1093/ecco-jcc/jjab076.396

[10] Neurath L , D’Amico F , Danese S . Emerging drugs for the treatment of moderately to severely active ulcerative colitis: review of phase II and III clinical trials. Expet Opin Emerg Drugs. 2023;28(1):28–42. 10.1080/14728214.2023.2186399 36876333

[11] Raine T , Danese S . Breaking through the therapeutic ceiling: what will it take? Gastroenterology. 2022;162(5):1507–1511. 10.1053/j.gastro.2021.09.078 34995533

[12] Em B , Jl S , Jacob J , Steiner CA , Stidham RW , Shannon C , et al. Efficacy and safety of dual targeted therapy for partially or non‐responsive inflammatory Bowel disease: a Systematic review of the literature. Dig Dis Sci. 2023:68. 10.1007/s10620-023-07837-0 PMC9942632 36807832

[13] Jairath V , Zou G , Wang Z , Adsul S , Colombel JF , D’Haens GR , et al. P588 Interim results from the randomised VERDICT trial to determine the optimal treatment target in patients with ulcerative colitis. J Crohn’s Colitis. 2023;17(Supplement_1):i716–i717. 10.1093/ecco-jcc/jjac190.0718

[14] Fenton CG , Taman H , Florholmen J , Sørbye SW , Paulssen RH . Transcriptional signatures that define ulcerative colitis in remission. Inflamm Bowel Dis. 2021;27(1):94–105. 10.1093/ibd/izaa075 32322884 PMC7737162

